# Dynamic changes in binding interaction networks of sex steroids establish their non-classical effects

**DOI:** 10.1038/s41598-017-14840-9

**Published:** 2017-11-01

**Authors:** Mónika Bálint, Norbert Jeszenői, István Horváth, István M. Ábrahám, Csaba Hetényi

**Affiliations:** 1 Department of Pharmacology and Pharmacotherapy, University of Pécs, Szigeti út 12, 7624, Pécs, Hungary; 2 Department of Biochemistry, Eötvös Loránd University, Pázmány Péter sétány 1/C, 1117, Budapest, Hungary; 3 MTA NAP-B Molecular Neuroendocrinology Group, Institute of Physiology, Szentágothai Research Center, Center for Neuroscience, University of Pécs, Szigeti út 12, 7624, Pécs, Hungary; 4 Chemistry Doctoral School, University of Szeged, Dugonics tér 13, 6720, Szeged, Hungary

## Abstract

Non-classical signaling in the intracellular second messenger system plays a pivotal role in the cytoprotective effect of estradiol. Estrogen receptor is a common target of sex steroids and important in mediating estradiol-induced neuroprotection. Whereas the mechanism of genomic effects of sex steroids is fairly understood, their non-classical effects have not been elucidated completely. We use real time molecular dynamics calculations to uncover the interaction network of estradiol and activator estren. Besides steroid interactions, we also investigate the co-activation of the receptor. We show how steroid binding to the alternative binding site of the non-classical action is facilitated by the presence of a steroid in the classical binding site and the absence of the co-activator peptide. Uncovering such dynamic mechanisms behind steroid action will help the structure-based design of new drugs with non-classical responses and cytoprotective potential.

## Introduction

**Figure 1:**
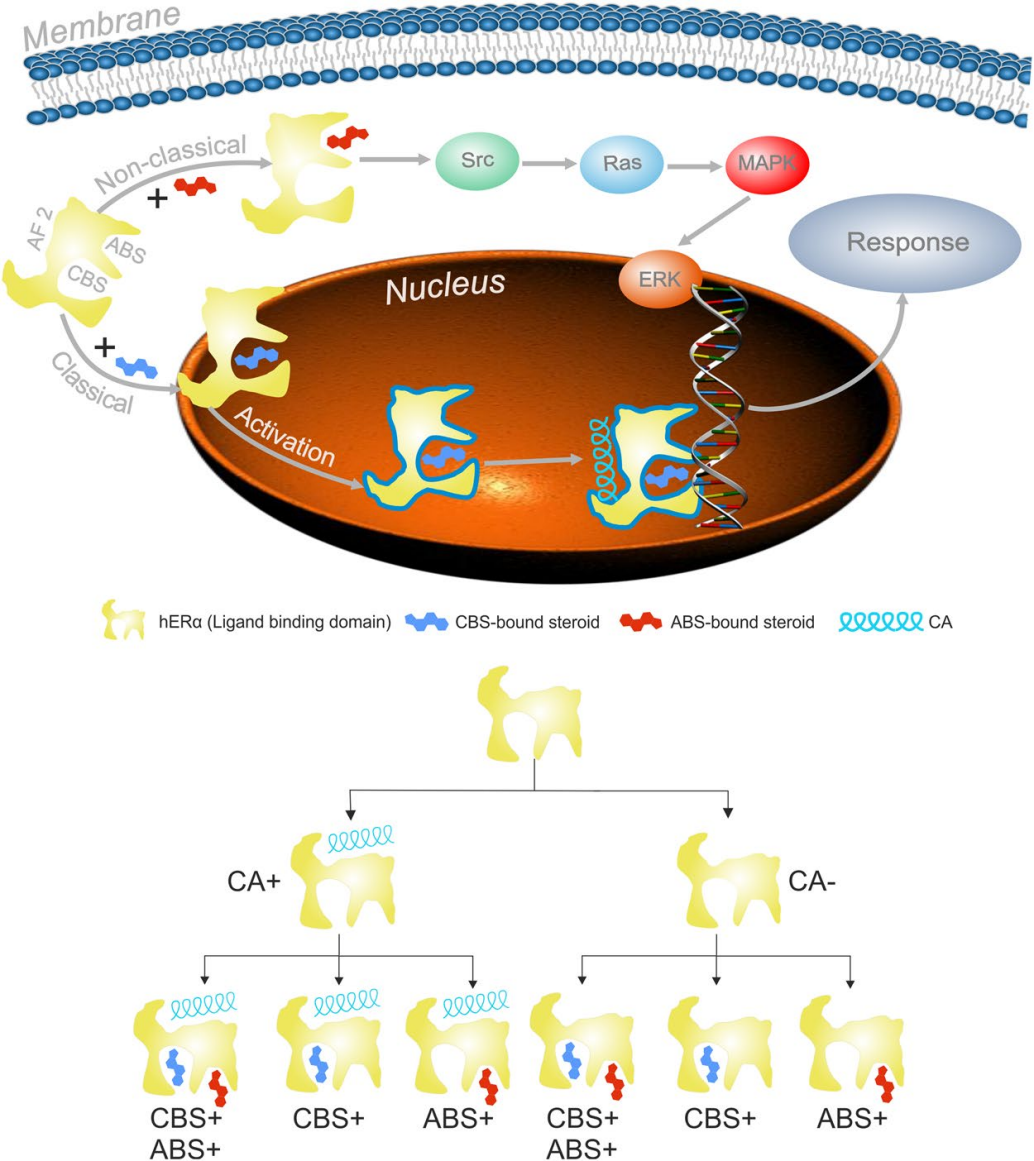
Effects of sex steroids in the cell (top) and their possible binding scenarios to human estrogen receptor alpha experimented in our study (hERα, bottom). Binding site of the co-activator (CA) is marked as AF2. Sex steroids can bind to classical (CBS) and alternative binding sites (ABS) as marked on the schematic representation of the ligand binding domain of hERα. In the classical pathway, activation of hERα by steroid binding to CBS is necessary for subsequent CA binding to AF2. In the non-classical pathway, steroid binding to ABS initiates signal transduction via Src, Ras proteins of the mitogen-activated protein kinase (MAPK) pathway.

Estrogens are responsible for a wide range of biological actions from the regulation of fertility to cytoprotection^1–3^. Gonadal 17β-estradiol (E2) has a remarkable neuroprotective potential^4^. Besides slow, classical, genomic effects^5,6^ (Fig. 1) E2 also exerts rapid, non-classical effects on intracellular second messenger molecules^7–10^, via estrogen receptors (ERs, Fig. 1).

Importantly, neuroprotection of E2 is attributed to such rapid actions^11–14^ and its binding to estrogen receptor alpha (ERα)^15^. Previously we have shown that a single dose of E2 as well as Activators of Non-Classical Estrogen-Like Signaling (ANCELS) such as estren-3α,17β-diol (EN)^16^ induce ERα-dependent neuroprotection via intracellular signaling pathways in neurodegenerative animal model^17,18^. The protective effect was also observed after traumatic brain injuries^4^ in rodents. Clinical studies showed that hormone replacement therapy with estrogen and progestin^1^ decreases the incidence of neurodegenerative diseases such as Alzheimer’s disease, but it also increases risks of stroke and breast cancer. However, structural dynamics of biding events establishing non-classical E2 action on ERs has not been fully elucidated. The lack of such details of molecular mechanisms of neuroprotective actions of estrogens hinders the exploitation of their therapeutic potential.

Estrogen binding to the classical binding site (CBS) of human estrogen receptor alpha (hERα) is well-explained by atomic resolution structures of the Protein Databank (PDB)^5,19^. The CBS is located between helices H3, H4, H6, H8 and H11^20^ (Supplementary Video S1) of the ligand-binding domain (LBD) of hERα, and it is known to mediate the slow, genomic actions of ligands, such as the native agonist E2 and antagonist 4-OH-tamoxifen selectively modulate gene expression^21^.

Besides slow, genomic actions (Fig. 1 top) ANCELS such as EN^22^, substance A and substance B^23^ exhibit weak transcriptional activity, selectively activating the non-classical E2 signaling as validated by functional assays^22,23^. Such non-classical actions of E2 on the signaling system have been known for more than forty years^24^. However, the underlying mechanism has not been understood due to the lack of atomic resolution structures of the complexes of effector ligands and ERs. An interesting study^25^ proposed an alternative binding site (ABS) of E2 and EN on hERα, further discussed by Norman and co-workers^26^, conveying the non-classical actions, analogously to vitamin D receptor^25^. The proposed ABS is located at the C terminus of H1 and N terminus of H3 helices, with a conserved R residue (R274 in vitamin D receptor and R394 in hERα) in the site. E2 binding to ABS^26^ does not directly alter gene expression, but rapidly activates the mitogen-activated protein kinase/extracellular-signal regulated kinase (MAPK/ERK) signaling pathway instead (Fig. 1, top)^8,9^.

Previous studies^25,27^ identified R394 and E353 as key E2-binding residues of ABS, located at the proximity of 3-hydroxyl group of E2, while the other, 17-hydroxyl group is oriented to R335^25^. From these results, a conformational ensemble model was constructed^26^ to explain the different behaviour of the nuclear and membrane associated forms of hERα. In this model, a “concurrent occupancy” was also proposed, when both ABS and CBS sites are simultaneously occupied by two copies of E2. However, the dynamics of simultaneous occupancy has not been investigated yet.

Besides ABS and CBS, there is a binding site for different transcriptional co-activator proteins. A conserved, LXXLL binding motif can be found in the amino acid sequences of these proteins^28^. Receptors are often co-crystallized with a peptide fragment of the co-activator (CA) protein containing the above conserved sequence bound to the activation function site 2 (AF2 site, Fig. 1 top part)^5,29,30^. In these structures, CA bridges between helices H3 and H12^20,31^ via hydrogen bonding at residues K362 on the H3 side and E542 on the H12 side. Furthermore, if E2 binds, and hER is activated (Fig. 1 top), the CA bridge fixes H12 in a position covering the E2-bound CBS^20,26^ and shielding it from the bulk solvent. Y537 plays an important role in the activation, and it was demonstrated that it is very prone to mutations (Y537S) which make the receptor resistant to estrogen antagonist drugs^30^. H3 residues E353, H356, M357 and W360 are proposed to form the ABS, and therefore, any perturbation of the conformation of H3 at these residues by CA can influence the binding of ligands to ABS, as well. Despite the importance of the above effects of the CA-bridge on E2 binding, the dynamics of the underlying mechanism, and the route of structural communication between the proposed ABS^25,26^ and CA has not been elucidated at atomic level.

Although the current cutting edge super-resolution imaging techniques such as single molecule fluorescence resonance energy transfer or stimulated emission depletion microscopy are capable to produce sequence of images in given time frame they have limited temporal (5 μs) and spatial (1 nm)^32,33^ resolution. Due to the limitations of current structure determination techniques^34,35^ investigation of the above questions is fairly challenging and “new techniques may be required to study the formation of such transient, though potentially biologically meaningful complexes” of sex steroids with hERα^16^. At present, molecular dynamics (MD) calculation is the only approach available for investigation of such real time binding events in a receptor-ligand system at atomic resolution. Consequently, several research groups apply MD calculations and present their results on conformational changes of various proteins^36–38^ and binding events of ligands^20,39–43^ at atomic resolution.

Accordingly, the present study also applies up-to-date, extensive MD calculations to investigate the real time changes of interaction networks of hERα and its ligands at atomic level. The structural dynamics of steroid binding was investigated at both ABS and CBS, taking into account the role of the CA, as well. For this, blind docking of E2 and EN to hERα was performed for an unbiased mapping of available sites. Subsequent MD of the docked complexes surrounded by several thousand of explicit water molecules was applied mimicking the natural dissociation route of the sexual steroids from hERα. The present study also aims at an MD-based elucidation of atomic resolution history of structural changes of ER accompanying non-classical steroid actions.

## Results and Discussion

### Interaction networks in the steroid-free receptor

**Figure 2:**
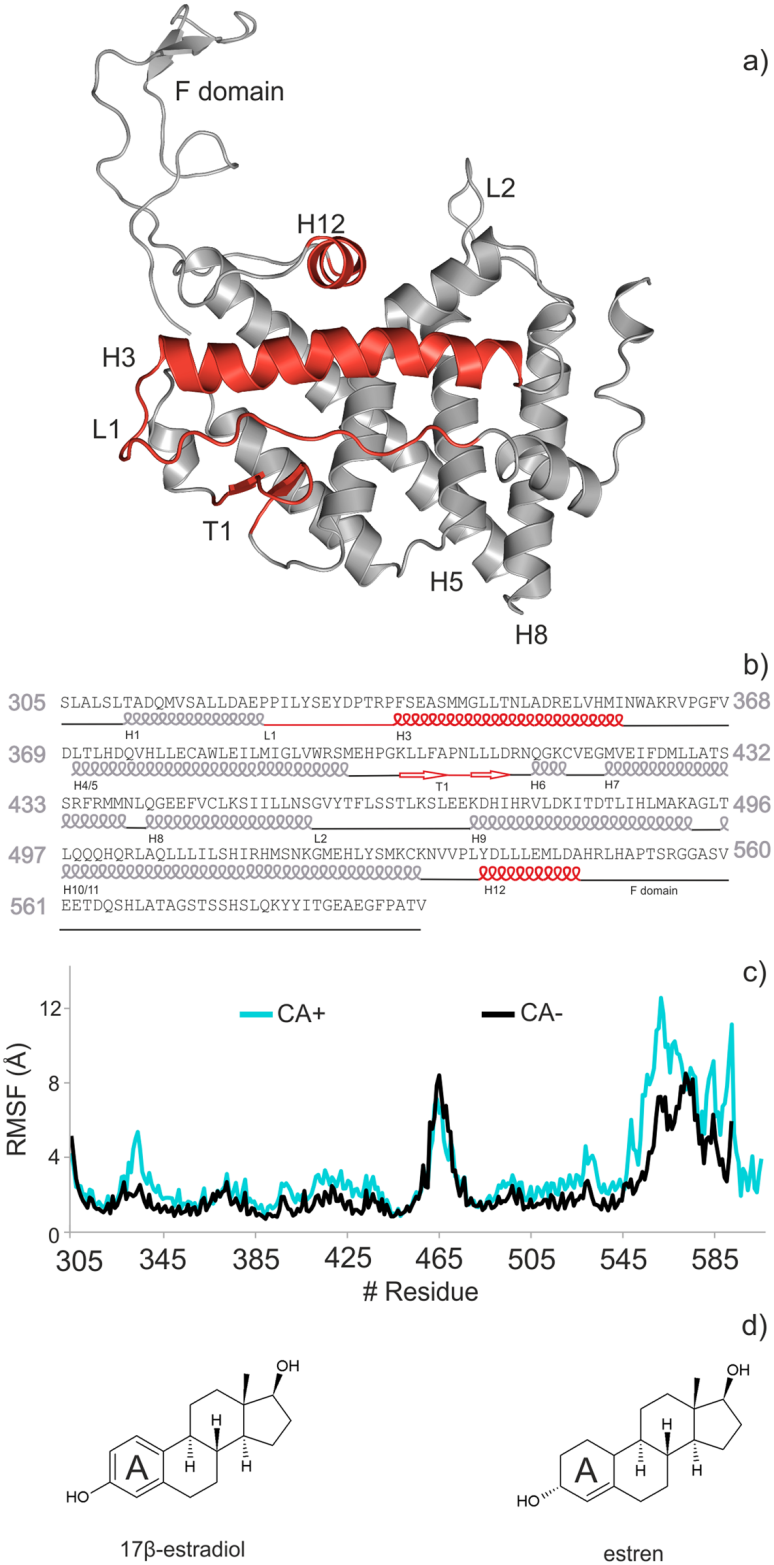
Molecules investigated in the present study. (**a**) The three-dimensional structure and the corresponding amino acid sequence (**b**) of ligand binding domain of human estrogen receptor alpha (hERα). Important helices H12, H3, loop L1, and β-turn T1 are highlighted in red. (**c**) Structural flexibility of hERα calculated as root mean squared fluctuations of all amino acid residues during the 1µs molecular dynamics simulations in co-activator bound (CA+) and unbound (CA−) forms of the steroid-free hERα. (**d**) Sex steroids 17β-estradiol, and an activator of non-classical estrogen-like signaling, estren.

To study the effect of CA binding on structural dynamics of hERα (Fig. 2a,b and Supplementary Video S1), both the CA bound (CA+) and free (CA−) structures of the steroid-free LBD were investigated (Fig. 2c) for comparison. The p160-type CA^44,45^ with crucial role in gene transcription was invloved in the present study. The C-terminus of the LBD was completed with a region called F domain extending the crystallographic structure using a modeling procedure described in Methods. In both cases, 1μs-long molecular dynamics (MD) calculations were performed to study the structural evolution of the LBD. Evaluations of the resulted trajectories showed (Fig. 2c) high root mean squared fluctuations (RMSF) of amino acid heavy atoms over the entire 1-μs domain at loops L1, L2, and in the F domain. Since loops are naturally flexible regions, and the F domain is a disordered region such fluctuations were expected. The flexibility of L1 can be explained mostly by its high exposition to the bulk. This loop is of high structural importance, as it has an indirect contact with the CBS through S329, and is also closely connected to helix H3, which is covering both the ABS and CBS (Supplementary Video S1).

**Figure 3:**
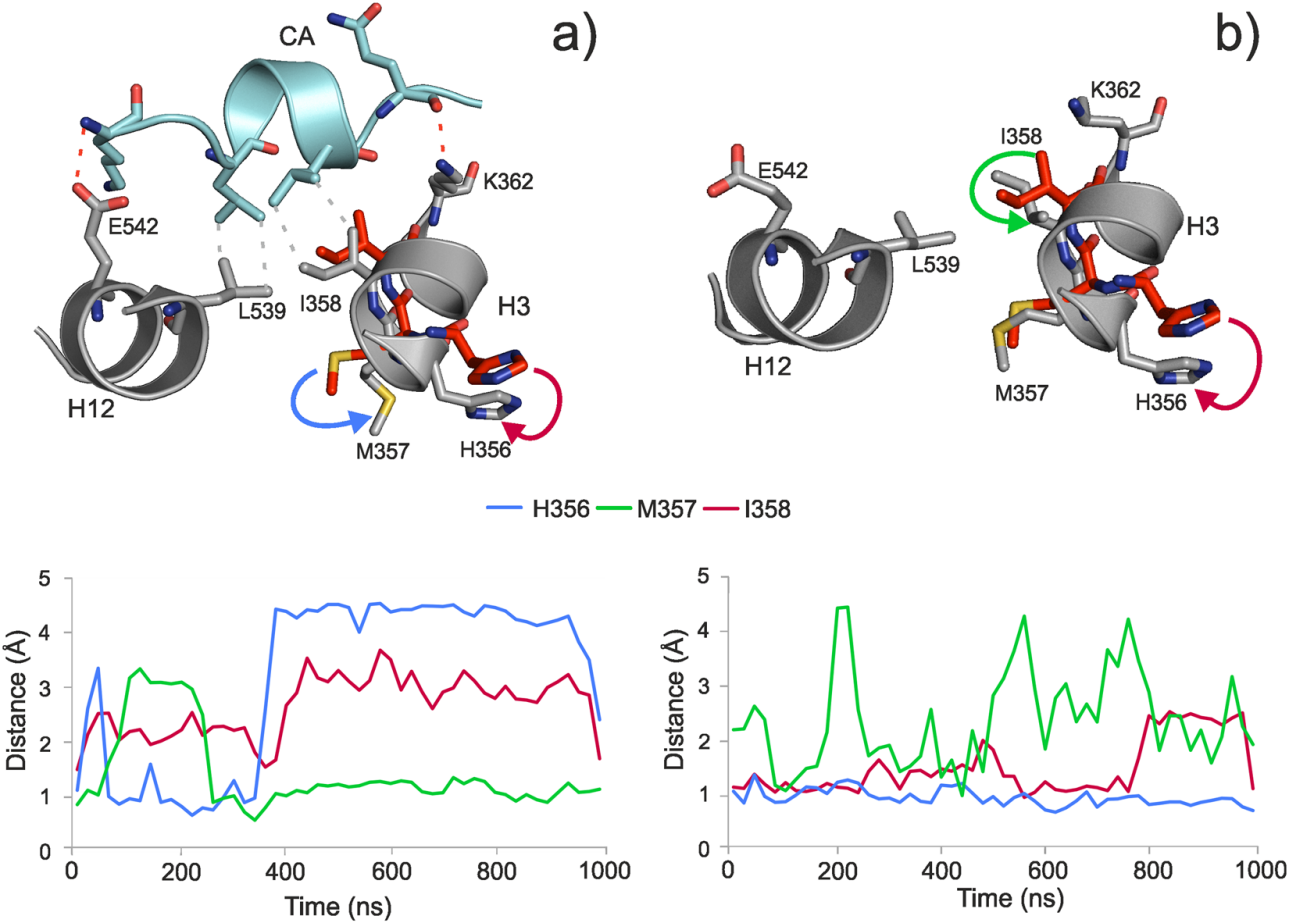
Detailed conformational changes at helices H3 and H12 of the human estrogen receptor alpha (hERα). 1 μs-long steroid-free molecular dynamics (MD) simulations were performed on co-activator bound (CA+, **a**) and unbound (CA−, **b**) hERα structures. The upper part shows conformation of important residues before (red) and after (grey) the MD simulations. CA is presented with cyan cartoon and sticks. A salt bridge, a H-bond (red dotted lines), and hydrophobic interactions (grey dotted lines) can be observed between CA and the helices (H12, H3) of hERα. In the bottom part, actual distances of M357, H356 and I358 from their initial positions are plotted during the MD simulation. Arrows on the upper parts have the same color codes as line charts on the bottom parts of (**a** and **b**).

Having MD results on both CA+ and CA− LBD structures, the influence of CA binding on the LBD was structurally analyzed paying special attention to the CA-connected helices H12, H3 and regions around the binding sites. Both termini of CA are connected to the LBD by salt bridges to E542 of H12, and by an H-bond to K362 of H3. In addition, CA forms hydrophobic contacts with I358 and M357 of H3 and L539 of H12 (Fig. 3a). The hydrophobic contacts with H3 are of particular interest, as I358 is in the vicinity of M357, which is part of the ABS. Therefore, comparison of their movement in CA+/CA− simulations may help to elucidate the mechanism of influence of CA on the process of ligand binding or dissociation to or from ABS. Accordingly, the movements of amino acids H356, M357, and I358 were quantified by calculating the distances between actual and initial positions of their side-chains (Fig. 3 bottom parts) along the MD simulations.

In order to maintain the hydrophobic interactions between the hERα, and CA (Fig. 3a top), I358 situated on H3 fluctuates between a distance of 2–3 Å measured from its initial position as a reference point (Fig. 3 bottom). The fluctuation (Fig. 3a bottom) is higher in the first part (0–400 ns) than in the second part of the simulation (400–1000 ns). The resulted 3 Å shift of I358 from its initial position causes the flipping of M357 into the ABS after 400 ns to further initiate the shift of H356 (Fig. 3a, bottom). In the CA− scenario (Fig. 3b top and bottom), it can be observed, that I358 highly fluctuates during the entire simulation. However, the above-mentioned shift of M357 into the ABS was not observed in the CA− scenario. Thus, the presence of CA can be perceived as a restricting factor, especially on M357. In contrast to M357, the orientation of H356 was not dependent on the presence or absence of CA at the end of the simulations. This can be explained by the contact between H356 (H3) and L327 (L1) through which L327 (L1) transfers its high mobility (Fig. 2c and Supplementary Video S1), to H356 (H3), then M357(H3). It was also found that in both CA+ and CA− simulations, H356 was oriented inside the ABS binding site by the end of 1µs simulation, but this switch occurs faster in the CA+ simulations (400 ns), than in CA− simulations (800 ns, Fig. 3 bottom parts) due to the movement of M357.

In the nucleus, sex steroids^45^ bind to the CBS activating hERα which results in the occupancy of AF2 binding site^46^ by CA (Fig. 1 top part). Such activation does not occur if hERα resides in the membrane and the AF2 site is left unoccupied. The membrane bound form of the estrogen receptor is involved^47,48^ in non-classical effects such as antiapoptotis^16^, cytoprotection, and neuroprotection^11^ Kousteni and colleagues have also reported rapid, non-classical effect of E2^16^, which require the extra-nuclear localization of the hERalpha, confirmed by confocal laser scanning microscopy studies^49^. The above-mentioned antiapoptotis is resulted by targeting^50^ an ABS outside the CBS of hERα. Fluorescence experimental studies^51^ also indicated the presence of ABS. Thus, ABS is linked to non-classical effects attributed to the membrane-bound form of hERα.

We found that ABS is available for ligand binding if AF2 is not occupied, otherwise it is dynamically blocked by both M357 and H356 side-chains. Thus, receptor dynamics at these two amino acids is responsible for the availability of ABS in membrane surrounding for certain ligands. In agreement with the herein presented results, experimental studies showed^16^ that E2, EN and other sex steroids are capable to produce non-classical effects^22^, occupying the ABS^27^. For this, sex steroids require extranuclear, membrane-bound localization of the estrogen receptor^22^, where the AF2 binding site is not occupied by CA.

### Binding sites of sexual steroids

**Figure 4:**
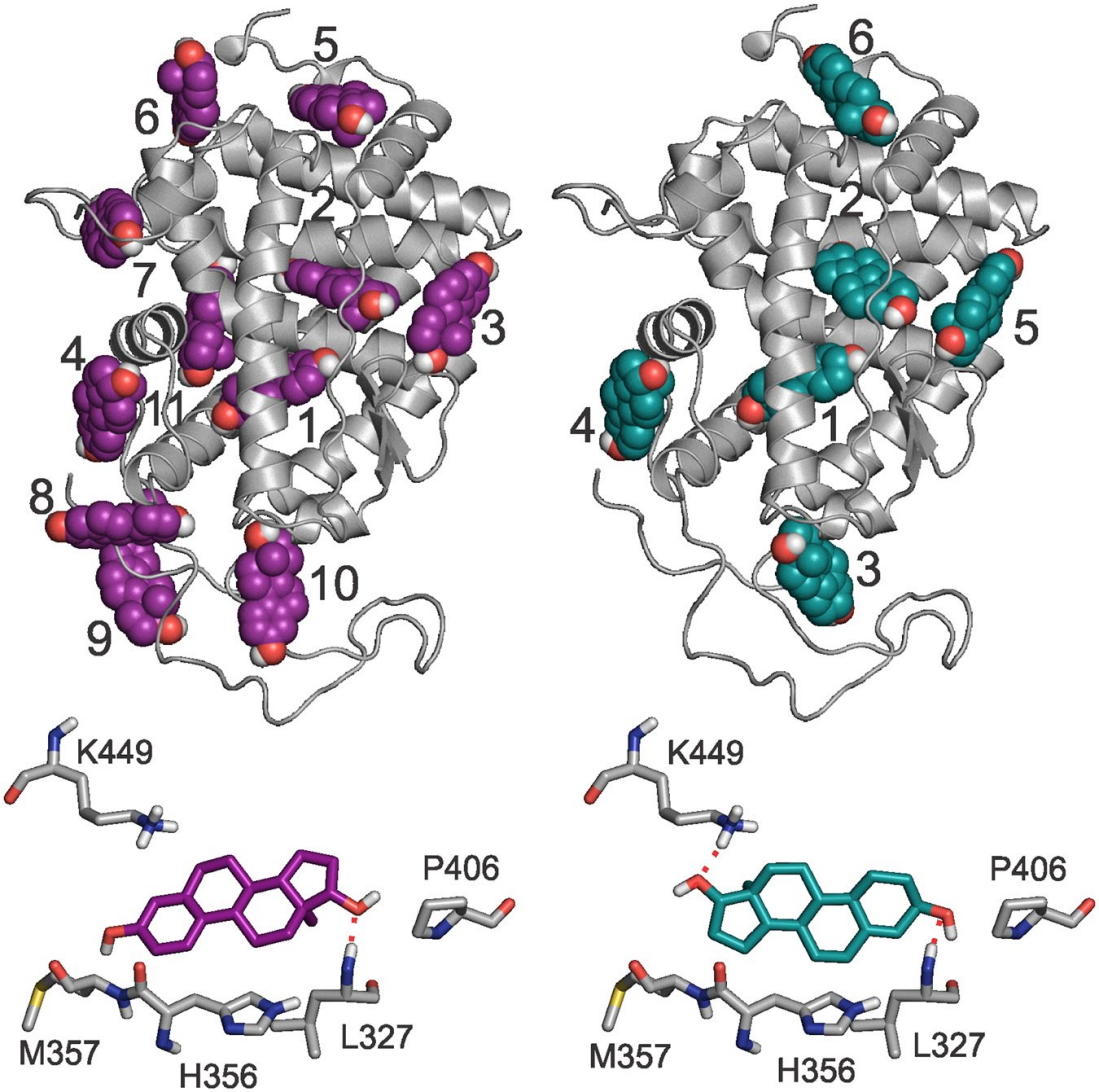
Results of blind docking calculations of steroids E2 (left) and EN (right) to hERα. At the top, cluster representant steroid conformations (spheres) and the corresponding rank numbers are shown. A small rank number corresponds to energetically favorable binding mode of the steroid. The receptor is shown as grey cartoon. At the bottom, a close-up of binding conformation of steroid E2 (magenta) and EN (green sticks) is shown in the ABS (Rank 2 in both cases). Neighbouring hERα residues are shown as sticks with grey carbon atoms.

Following structural dynamics investigations on the steroid-free receptor, a complete exploration of binding sites of sex steroids was performed on the entire surface of the apo LBD. Blind docking^52–54^ was used for the search as this method does not require previous knowledge of the location of the binding sites. A representative structure of LBD was produced by MD simulation with subsequent clustering (Methods) and used as a target in the blind docking calculations (Fig. 4). The target structure was validated by blind docking of E2 (Fig. 4, magenta). The docking result was compared with the crystallographic ligand conformation in the CBS (Supplementary Fig. S1). One-hundred blind docking trials were performed with random initial positions of E2 around the target. The results were evaluated as described in previous works^52,53^. Briefly, the docked steroid copies were clustered and ranked by energy, resulting in a list of explored binding sites and ligand poses with the strongest steroid-site interaction in the first rank. Besides E2, blind docking of EN (Fig. 4, teal) was also performed on the LBD. From the blind docking calculations 11 ranks were identified for E2 and 6 for EN (Fig. 4, Supplementary Table S1).

The CBS was found in the first rank of blind docking by both steroids. Reproduction of the binding mode of E2 in the CBS was successful as a root mean squared deviation (RMSD) of 2.1 Å (Supplementary Fig. S1) was measured between the heavy atoms of the blind docked and crystallographic (reference) steroid conformations. Such a good fit of the docked E2 to the experimental conformation shows that the target LBD structure is valid and blind docking predictions provide accurate results at atomic resolution. Analysis of docked molecules in CBS revealed that binding modes of E2 and EN are very similar to each-other (Supplementary Fig. S2). Both steroids occupy the same orientation with H-bonds formed between 3-hydroxyl of E2 and EN and hERα residues (F404, E353, and R394). Topologically, CBS is separated from the bulk by loop L1, ß-turn T1, H3 and H12. At the same time, structural differences between the steroids influence their hydrophobic interactions with the amino acids in the surroundings (Supplementary Fig. S2). For example, aromatic ring A of E2 (Fig. 2d) forms a perpendicular π-stacking with F404 situated on T1. The lack of aromatic ring in EN results its increased flexibility and weak hydrophobic interactions with F404, if compared to the π-stacking, observed at E2. This could also be part of the reason, why E2 is considered primarily as a CBS-binding ligand^26,44^ and is selective for the classical pathway^16^.

The ABS was found in the second rank during blind docking of both E2 and EN in a region proposed by previous studies^26,27^. ABS is located between H8 and H3 in the vicinity of the CBS (Supplementary Video S1). Exposition of ABS towards the bulk is higher than that of CBS as it is covered only by the highly flexible L1 (Fig. 2a). Similarly to the “ensemble model” of previous studies^26,27^, the BD calculations showed that R394 and E353 separate the two sites (Supplementary Fig. S3). Furthermore, EN is bound to the ABS, with its 3-hydroxyl group oriented towards R394, which also agrees with previous studies^25,26^. Lipophilic residues (P324, L327, M357, W360, I386, P406) dominate this site, K449 is the only amino acid with polar side chain. Comparing the binding modes of the two analyzed steroids (E2 and EN) to the ABS, a head-tail swap can be observed between them (Fig. 4, bottom). Accordingly, a hydrogen bond is formed with the backbone amide of L327 with different groups of the steroids (17-hydroxyl of E2, and 3-hydroxyl of EN). In addition, 17-hydroxyl of EN forms another hydrogen bond with K449. This bond was not observed in the complex with E2. The H-bond with the backbone amide of L327 is common for the two ligands. As L327 is on loop L1 it is exposed to the bulk, mobile and susceptible to the thermal motion of the surrounding water molecules (see also Section Interaction networks in the steroid-bound receptor and results on simulations with different velocity distributions). At the same time, the second H-bond specific for EN is formed with H8, buried in the pocket, inaccessible from the bulk stabilizing the interaction of EN with the LBD at the ABS. Concerning the location of ABS and CBS the results are in good agreement with previous studies^25–27^. A previous comparison of the binding interaction energies of E2 and EN produced by manual docking^25^, showed that binding of EN is stronger to the ABS than that of E2 (Table 1). For the CBS, an opposite trend was observed (Table 1). Other docking^25,27^ studies also confirmed E2 selectivity towards CBS. Experimental binding studies demonstrated^22,55^ that E2 has a higher affinity towards ERα than EN. *In vitro* experiments^22,55^ showed that E2 plays a role in classical effects associated with its CBS^50,51^ binding. At the same time, despite the moderate binding affinity of EN^23^ *in vivo* studies^22^ also confirmed that it has a selectivity towards the non-classical pathway, lacking an effect on the reproductive organs which was confirmed by histological analysis of the uterus, and did not stimulate transcription of the C3 gene in the uterus^22^. In the present study, interaction energies were calculated using the docked and energy-minimized ligand structures. The differences in the energy values show good agreement with those obtained in previous docking (Table 1) and the affinity/selectivity preferences demonstrated by the above-mentioned *in vitro* and *in vivo* experimental studies.

**Table 1:** Interaction energies of sexual steroids with hERα (kcal/mol).

Ligand	Present study		Mizwicki *et al*. 2004	
	ABS	CBS	ABS	CBS
EN	−37	−33	−66	−61
E2	−32	−35	−61	−66

Table 1 shows that EN binds 4 kcal/mol stronger to ABS than to CBS. At the same time, the binding of EN to ABS is 5 kcal/mol stronger than that of E2. This is in agreement with the above structural findings, and also with previous results^25^, showing that EN has a larger affinity to ABS than CBS. These results suggest different binding modes at ABS and CBS which is consistent with the structural observations described above (Fig. 4).

All-in-all, for the top two ranks blind docking gave consensus results identifying the binding sites of both steroids as the CBS and the ABS, respectively. Both steroids bind to both sites with significant interaction energies, with E2 a classical effector on CBS, and EN preferring ABS as a non-classical effector^26^. In Rank 3 and beyond, steroids found different sites without a consensus result. Notably, binding of E2 to CBS had been precisely described^5,20^ and the position of ABS was proposed in previous studies^25,27^. However, steroid binding to ABS has not been fully characterized. Here, atomic resolution structures of the complexed sites with both investigated ligands bound to ABS were provided (Fig. 4), highlighting crucial amino acids, for non-classical activity, and the binding difference between them. Moreover, binding mode of EN to CBS was also provided (Supplementary Fig. S2) and analyzed. Atomic resolution complex structures from the above blind docking calculations were piped in the investigations of the next Section dealing with the molecular dynamics of interaction networks of steroid binding.

## Interaction networks in the steroid-bound receptor

### Interaction dynamics

To effect the transcriptional activity in the classical, genomic pathway, a “long-lived”^16^ steroid-CBS contact is needed in order to produce the specific conformational changes of hERα. At the same time, steroid ligands form “transient complexes” with the ABS, via a brief association to hERα in the non-classical pathway. However, investigation of such rapid effects of the non-classical pathway requires new approaches and techniques^16^.

In the present study, we apply molecular dynamics calculations of the steroid-bound hERα surrounded by several thousand (explicit) water molecules. To investigate the interaction dynamics, docked steroid-bound receptor structures were adopted from Section Binding sites of sexual steroids as starting points. Besides singly occupied binding sites, additional complex structures were constructed (Methods) with both ABS and CBS simultaneously occupied for both EN and E2. All versions were produced both in the presence and absence of CA which yielded altogether twelve different complexes for the two sexual steroids (Fig. 1, bottom). For all complex structures, five parallel 100-ns-long MD calculations were performed to follow their trajectories. Thermal dissociation of the steroid ligands was expected by acquiring kinetic energy from its water and protein surrounding. The calculations were repeated five times using different initial velocity distributions resulting in a total of 6 µs MD calculation. Applying more than one starting initial velocity distribution for a starting structure is important to obtain statistically relevant, unbiased conclusions. In other words, five, independent dissociation trials were performed resulting in five, independent dissociation trajectories of the steroids in all twelve complexes.

**Figure 5:**
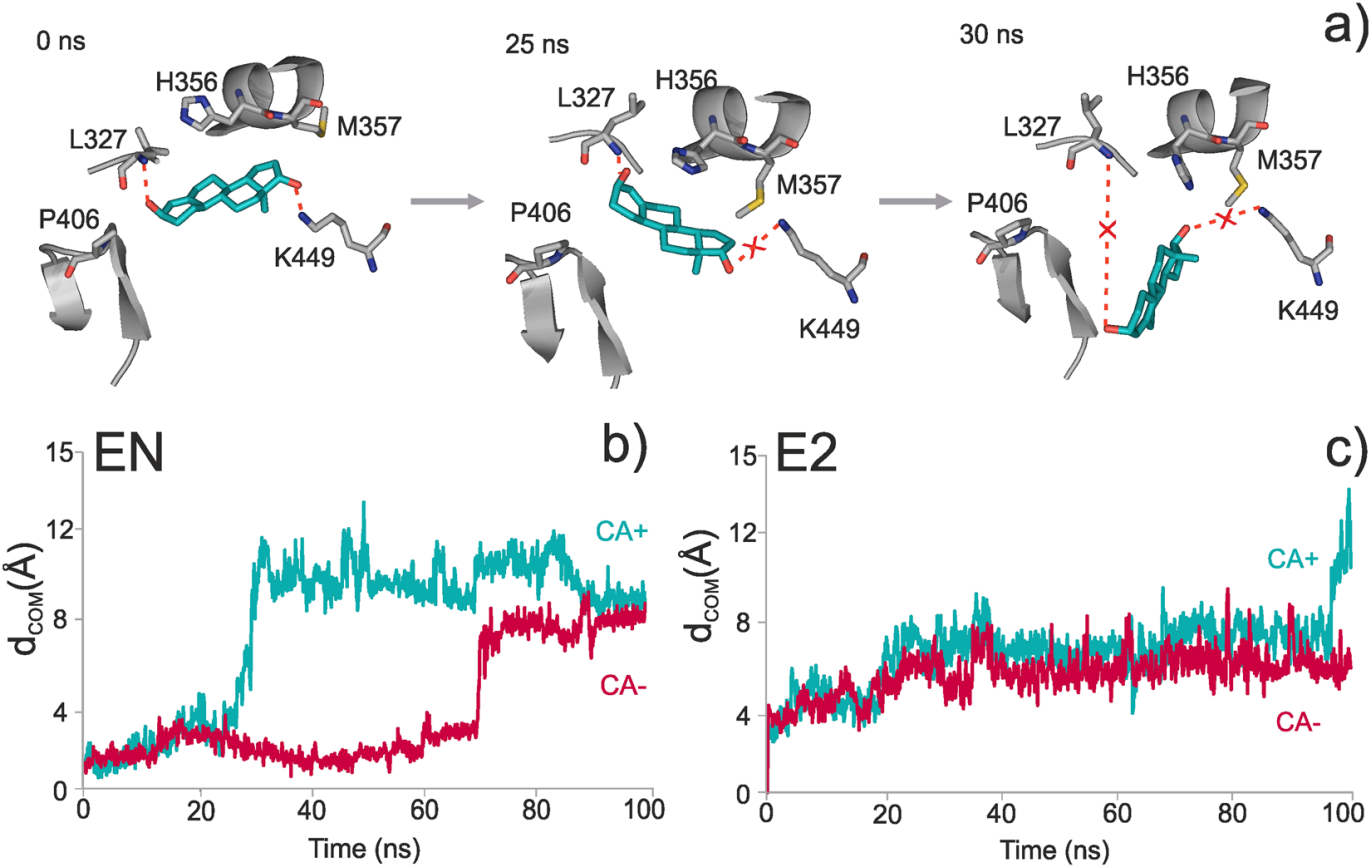
Dissociation of the steroids from the ABS. (**a**) Snapshots of the 100-ns-long molecular dynamics (MD) simulation of EN dissociation from the ABS. The simulation started from a CA+/CBS+/ABS+ starting complex. Disruption of H-bonds with K449, and L327 can be observed at 25 and 30 ns, respectively. Migrations of steroids EN (**b**) and E2 (**c**) out of ABS are represented as actual distances of their center of mass (d_COM_) measured from their bound, starting position inside ABS. Evaluations of simulations both with (CA+) and without (CA−) the co-activator are shown. In the case of EN (**b**), an abrupt increase of d_COM_ can be observed, at 30 ns (CA+) and at 70 ns (CA−). Thus, the presence of CA promotes the dissociation of EN from ABS. Dissociation of E2 shows a different picture (**c**), as its d_COM_ increases in a stepwise manner, without fast jumps in the starting period of the simulation. This is due to the lack of strong, directed interactions between E2 and ABS.

From the dissociation trajectories (Fig. 5a, Supplementary Video S2), residence frequency (RF) values were calculated to quantify kinetic stability of the complexes in each trial of Fig. 1 (bottom). In drug discovery, assessment of kinetic stability described by the residence of a ligand in the binding site is crucial factor similarly to thermodynamic stability^56,57^. To calculate RF, the movement of the ligand was described by the distance between the centre of mass (d_COM_) of its actual and starting positions at each time frame during the simulation time resulting in a COM-plot. The RF value of a binding site was directly obtained (Equation 3, Methods) from the COM-plots (Fig. 5bc) using a d_LIM_ = 5 Å for dissociation limit.

Results of the merged trajectories of a total of 500 ns simulation time per trial are listed in Tables 2 and 3. Per-trial and RMSD-based evaluations are presented in Supplementary Tables S2–S5. In the present study, the theoretical upper limit of RF was 10.0 ns^−1^, which corresponds to the highest kinetic stability. The mean CBS RF values of E2 and EN (last column in Table 2, average of first four columns), are 10.0 ns^−1^ and 9.0 ns^−1^, respectively. Experimental results are in agreement with our calculations (Table 2) affirming that E2 has a stronger affinity to CBS than EN^22,25^. Results in Table 1 are also in line with a key review by Norman and co-workers^26^ presuming that steroids such as EN and E2 could have different “fractional occupancies” in the ABS and CBS pockets. Whereas both ligands show good binding stability at the CBS, a drop in RF values can be observed at ABS (Table 3) if compared with those at CBS (Table 2). In the case of ABS, the mean RF of EN is markedly higher than that of E2.

**Table 2:** Residence frequencies of the steroids in CBS (ns^−1^).

CA	+		−		Mean (SD)
ABS	+	−	+	−	
EN	9.3	9.1	10.0	8.4	9.2 (0.7)
E2	10.0	10.0	10.0	10.0	10.0 (0)
Mean (SD)	9.7 (0.5)	9.6 (0.6)	10.0 (0)	9.2 (1.1)

**Table 3:** Residence frequencies of the steroids in ABS (ns^−1^).

CA	+		−		Mean (SD)
CBS	+	−	+	−	
EN	2.6	8.0	7.2	6.5	6.1 (2.4)
E2	1.8	2.9	5.1	2.3	3.0 (1.5)
Mean (SD)	2.2 (0.6)	5.5 (3.6)	6.2 (1.5)	4.4 (3.0)	

For structural interpretation of the results in Tables 2 and 3, representative individual trajectories were selected with RFs closest to that of the merged trajectory (bold in Supplementary Tables S2–5). As it was described in Section Binding sites of sexual steroids, EN has H-bonds with both hydroxyl groups, and is stabilized in the ABS at its both ends (Supplementary Video S2 and Fig. 5a). The two H-bonds are formed at the entrance with L327 of loop L1, and K449 of H8 helix, at the bottom of the pocket. Loop L1 is highly exposed to the bulk having a susceptibility to the thermal motion of the hydrating water molecules and it tends to pull out EN from the binding site. At the same time, forming an H-bond with K449, H8 acts as a counter balance and keeps EN in ABS. If the H-bond with K449 is broken, EN will be easily pulled out towards the bulk by the loop. After the breakage of the H-bond between EN and K449, a series of conformational changes are initiated by L327. Firstly, as L327 interacts with the side chain of H356 through hydrophobic interactions and H356 starts to move towards the ABS binding site, as fluctuation of L1 intensifies. Secondly, a conformational change is induced on M357 by H356. Here, the side chain of M357 flips into the ABS binding site, similarly to the apo simulations (see Secion 1). As M357 flips inside de binding site, sterically perturbs EN leading to its expulsion from the site. The above conformational changes were not observed in case of E2, and therefore, no role can be attributed to M357 in its dissociation. As the H-bond with K449 is missing in case of E2, the above described counter balancing effect does not take place. Hence, E2 is pulled out more easily than EN from ABS by the thermal motion of the loop.

**Figure 6:**
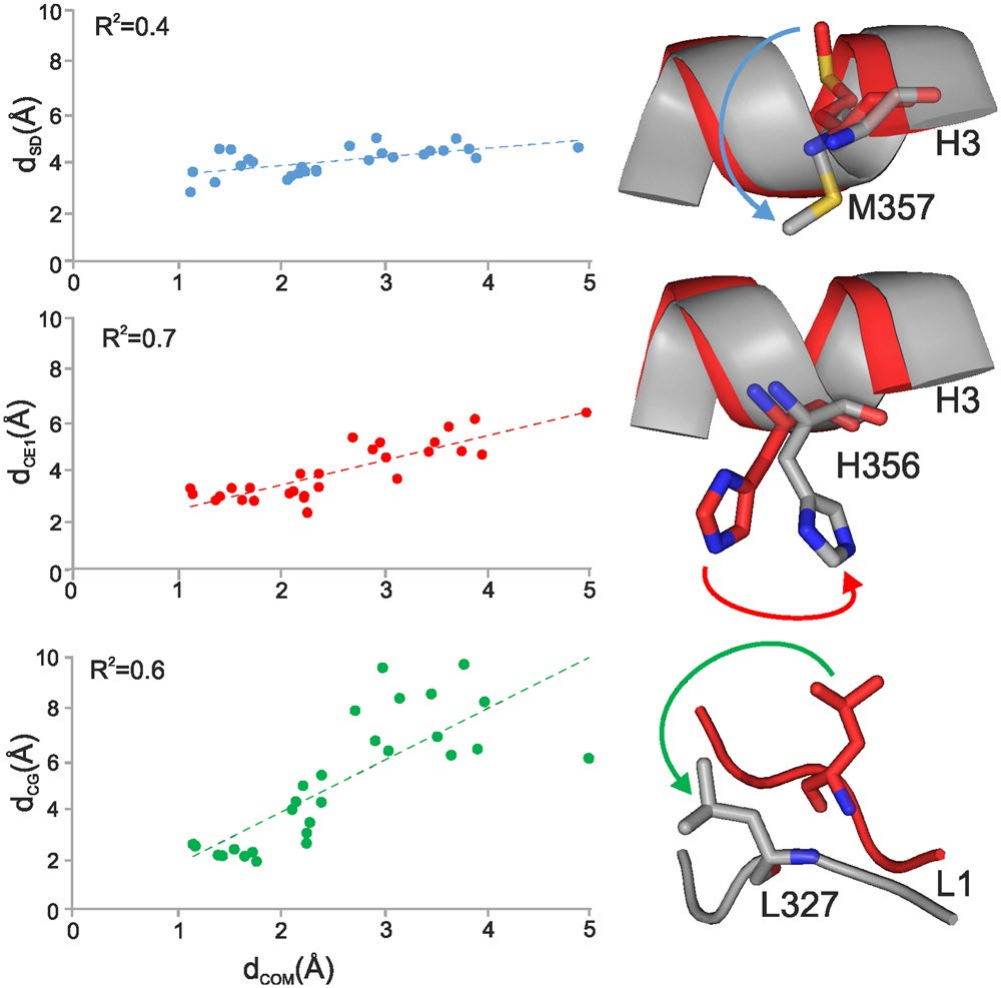
Correlation of movements of residues M357, H356, L327 with dissociation of EN. The movements of the residues are expressed as the distances of SD (M357), CE1 (H356) and CG (L327) atoms from their initial positions. Dissociation of EN is measured by d_COM_, the distance of the center of mass of the ligand, measured from its initial position. The importance of M357, H356, L327 in the dissociation of EN is indicated by the above obtained correlations. Correlation plots contain points until dissociation (d_COM_ < d_LIM_). The structural representations on the right correspond to d_COM_ = d_LIM_. For simplicity, the points represent average distances calculated as described in Methods.

The above analyses of the simulation trajectories highlighted that the conformational changes of hERα (Supplementary Video S2 and S3) have crucial role in the dissociation process of EN. In order to quantify the relationship between conformational changes of the receptor and dissociation of EN, d_COM_ was correlated with the movement of three residues (L327, H356, and M357) in the d_COM_ < 5 d_LIM_ interval. Correlation results are shown for the CA+/CBS+/ABS+ (Fig. 6) case with representative residue movements. Notably, similar correlations were observed for the CA−/CBS+/ABS+ case (Supplementary Fig. S4), as well. The obtained correlations show that all three proposed residues are important in inducing EN dissociation. Due to their characteristic interaction networks there is a considerable difference in the dynamics of the three side-chains (Fig. 6). While M357 enters the ABS, which results in pushing EN out of its binding pose, L327 exerts a pulling effect on EN from the other side. H356 continuously fluctuates rotating inside the ABS.

In this Section, dissociation mechanisms of sexual steroids from ABS and CBS were uncovered by extensive molecular dynamics calculations. Differences in binding affinities^16,25^ (Table 1) and kinetic stability (Tables 2 and 3) of steroid-hERα complexes was correlated with the differences in the dynamics of the corresponding interaction networks.

### Effects of co-binders

The steroid-free MD simulations uncovered an interference between ABS and AF2. It was found that the ABS is available for ligand binding only if AF2 is not occupied by CA (Section Interaction networks in the steroid-free receptor). The results of Table 3 also show that occupancy of ABS is influenced by the presence of other ligands co-bound to hERα. Binding of CA to site AF2 or an additional steroid molecule to CBS has considerable effect on steroid binding to ABS (Table 3). In order to investigate the structural background of these effects, we examine how CA affects the binding dynamics of E2 and EN to hERα.

In the CA− scenario, remarkably high stability of E2 and EN binding to ABS was found especially if an additional E2 or EN copy was present in the CBS (CBS+, third column in Table 2). This situation is of particular importance as non-classical effects happen in the absence of CA (Section Interaction networks in the steroid-free receptor). Various experimental studies have suggested that fast, non-classical activity of streoids is exerted by their binding to the ABS^16,50^. It is also known that binding to ABS is not probable in the presence of CA^30,46,58^. Consequently, the presence of CA (CA+) would hinder steroid binding to ABS and facilitate dissociation. Our MD approach allowed the investigation of such a non-natural CA+ situation and the analysis of the reasons of the hindering effect of CA binding to AF2, as well. This finding is consistent with the effect of CA over the ABS binding site (Section Interaction networks in the steroid-free receptor) where the effect of CA bridge connecting helices H3 and H12 was demonstrated. As both helices are very close to the binding sites, they interfere with the CA bridge and ligand binding. CA binds to the LBD via ionic and hydrophobic interactions with H3 at M357 and I359, which are part of the ABS. It was also demonstrated (Section Interaction networks in the steroid-free receptor) that M357 tends to occupy ABS in presence of CA. The same mechanism was observed also in the ABS+ simulations, but only in case of EN, which indicates a dependency on the ligand type (see also Section Interaction dynamics, Fig. 5). If CA is present, M357 tends to move towards the center of the ABS, and I359 assists this process providing a steric restraint and keeping a hydrophobic contact with L693 of CA. On the other hand, if CA is missing from the above interaction networks, its influence on I359 and M357 is not there. Thus, I359 can move freely, and therefore, M357 can maintain its orientation towards the bulk, and it does not influence the stability of EN binding. All-in-all, CA changes the dynamic interaction network of the ABS leading to kinetic stability differences presented as RFs in Table 3. Although H356 has no direct contact with CA it also plays an important role in the dissociation mechanism as it is in the vicinity of M357, and also occupies the ABS promoting the dissociation of EN (Fig. 5a and Supplementary Video S2).

The above structural effects are also reflected by the velocity of EN during the dissociation process from ABS as calculated from the COM-plot (Fig. 5bc). The overall dissociation velocity of EN increased from 0.11 to 0.25 Å/ns (Supplementary Tables S6 and S7) in the CA+ case, due to the described destabilizing effect of CA binding to hERα. The dissociation process of EN can be divided into an initial (d_COM_ ≤ d_LIM_) and a terminal (d_LIM_ < d_COM_ ≤ 10 Å) phase. Velocity of EN in the terminal phase is larger than it was in the initial phase, which is specific to EN. EN has higher v_2_ values than E2 suggesting that final dissociation of E2 from ABS occurs slower than in case of EN. The characteristic, abrupt movement of EN in the terminal phase can be explained by the sudden of breakage of the second stabilizing H-bond, the one with K449.

**Figure 7:**
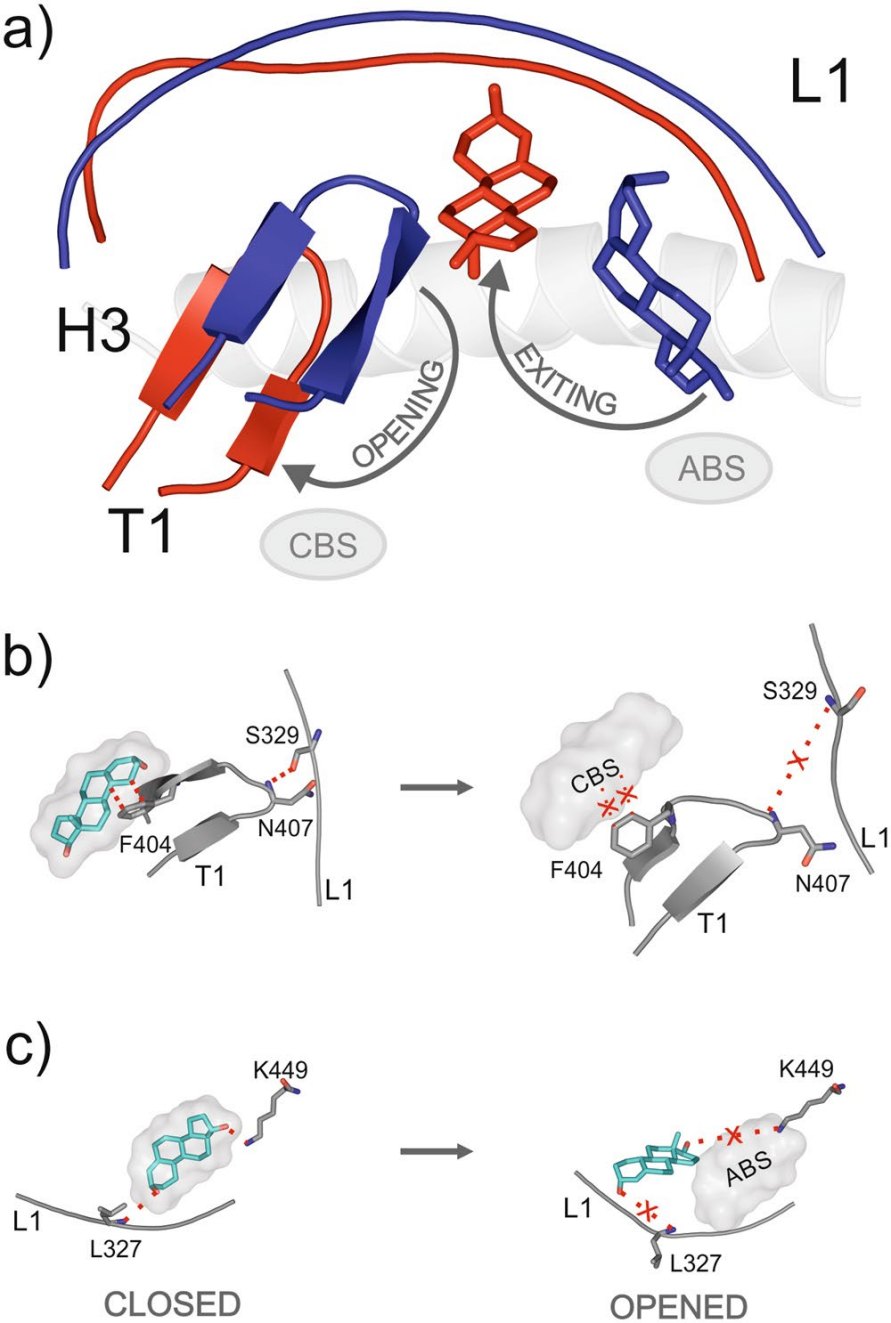
Dynamics of the flickering gate. (**a**) The three-dimensional structure of the flickering gate is composed by T1, L1, and H3 structural elements. Its closed (blue) and opened (red) states show considerable differences at T1 and L1 conformations. EN is represented with blue (ABS-bound) and red (unbound) sticks. (**b**) The absence of EN (sticks with green cartoon) from the CBS (grey cloud) results in the breakage of N407 and S329 in the opened state, releasing L1. (**c**) Consequently, L1 will not maintain its conformation necessary for the interactions with EN, which will readily leave ABS. (**b,c**) All atom coloring is used for sticks of EN and target residues (grey carbon). Red dotted lines highlight H-bonds, and hydrophobic interactions. Interactions present in the closed state, and absent in the opened state, are marked with red cross.

The effect of the presence of an additional steroid molecule in the CBS (CBS+) is coupled to that of the absence of CA and this CA−/CBS+ case shows the highest stability of the ABS-bound ligands (Table 3). To understand the effect of occupancy of the CBS simulations on EN were analysed for both CBS+ and CBS− cases (Supplementary Table S3, seed 1). Three structual elements T1, L1 and H3 were of particular interest, in analyzing the stabilizing effect of CBS over the ABS. These elements can be considered as parts of “flickering gate” (Fig. 7a and Supplementary Video S3) as proposed by a previous study^25^. T1 plays the role of the flickering wing, whereas L1 and H3 constitute the stable frame of the gate. Our calculations show that the gate is closed when CBS is occupied (Supplementary Video S3, blue), and opened when CBS is unoccupied (Supplementary Video S3, red). When EN binds to the CBS it is able to keep the “flickering gate” in a closed state, as it interacts with T1 (flickering wing) via a hydrophobic interaction with F404 (Fig. 7b). Therefore, in the closed state, stabilization of T1, by EN in the CBS, will further maintain an H-bond between T1 (N407) and L1 (S329). Stabilized by this H-bonding between T1 and L1 (Fig. 7b), L1 becomes less flexible, and its rigidity will further increase RF of EN in the ABS (Fig. 7c). This happens as L1 binds to EN in ABS via L327 (Fig. 7). See also Section Interaction dynamics showing that the movement of L327 correlates with ligand dissociation (Fig. 6). We found that the flickering gate adopts an opened state if CBS is not occupied (Fig. 7b). This is a consequence of the lack of hydrophobic interaction between T1 (F404) and the CBS-bound EN. The H-bonding between L1 (S329) and T1 (N407) becomes disrupted, and therefore, flexibility of L1 increases. As discussed above, a flexible L1 promotes dissociation of EN from ABS and lowers the corresponding RF (Table 3).

The above dynamic interaction network, especially between CBS, T1, L1, and ABS describe the working mechanism of the “flickering gate”^25^. Beside providing a detailed description of the opened and closed state of the gate, we were also able to detect two dissociation pathways of EN during exiting ABS. The first dissociation pathway towards F404 and P406 of T1 is shown in Supplementary Video S3, and the second pathway towards P323 of L1 can be followed in Supplementary Video S2. Both dissociation pathays require the opened state of the “flickering gate”^25^. In addition to the kinetic stability data of Tables 2 and 3, MD allowed the above in-depth analyses of changes of interaction networks at the ABS. The present approach provides a structural background of stability differences pointing to key residues of hERα affecting non-classical steroid action.

## Conclusions

In the present study, elucidation of structural dynamics of non-classical effects of sex steroids was presented. Both classical and alternative binding modes were exhaustively mapped on the ligand-binding domain of human estrogen receptor alpha. Kinetic stability of the steroid –receptor complexes was investigated by molecular dynamics calculations. Real-time investigations of the complete interaction network at atomic resolution pointed to key residues of steroid binding mechanism. We showed how steroid binding to the alternative binding site of non-classical action is facilitated by the presence of a ligand in the classical binding site and the absence of the co-activator peptide. Uncovering such dynamic mechanisms behind steroid action will help the structure-based design of new drugs with rapid, non-classical responses.

## Methods

### Steroid-free systems

#### Selection of target structure

There are 137 hERα LBD entries available in the Protein Databank (PDB, Supplementary Table S8) and among them structure 3q95 has the most amino acids solved with a good resolution of 2.05 Å. The 3q95 structure is co-crystallized with the native ligand (estriol) and CA. As 3q95 is the most complete structure, it was chosen to represent hERα LBD. The ligand-free hERα (2b23) is also available (Supplementary Table S8) and superimposing 2b23 and 3q95 on their backbone atoms with PyMol^59^ has an excellent overall structural fit quantified by a root mean squared deviation (RMSD) of 0.5 Å. The RMSD was calculated between the two conformations according to Equation (1).

1 $${\rm{RMSD}}=\sqrt{\frac{1}{{\rm{NH}}}\sum _{{\rm{i}}=1}^{{\rm{NH}}}{| \vec{{\rm{C}}{1}_{{\rm{i}}}}-\vec{{\rm{C}}{2}_{{\rm{i}}}}| }^{2}}$$

where NH is the number of heavy atoms, C1 and C2 are space vectors of the i^th^ heavy atom of conformations 1 (C1) and 2 (C2), respectively.

Secondary structure prediction was performed on the amino acid sequence of the missing F-domain, the sequence was accessed from UniProt with accession ID of P03372, multiple sequence alignment was performed with Clustal Omega^60^. Prediction was performed on the PsiPred server^61^, with the last two amino acids from X-ray structure added, to facilitate the fitting onto the protein after MD. Based on this prediction, the tertiary structure of the polypeptide chain was modelled with Tinker and equilibrated by a 10-ns-long molecular dynamics simulation. After equilibration, further 100 ns, unrestrained MD trajectory was generated for production (see next Section for details). After clustering, the representative structure of the C-terminal region, was merged with both X-ray structures of HERα (3q95 and 2b23) and these extended proteins were used throughout this study.

Both the ligand free and ligand bound PDB entries are appropriate representations of the LBD structure as E2 and estriol do not induce significant changes in the protein structure. In Section Interaction networks in the steroid-free receptor, the extended 2b23 was used, the holo simulations of Section Interaction networks in the steroid-bound receptor were performed with 3q95. The RMSF plot of 3q95 (1 µ simulation, without ligand, Supplementary Fig. S5) shows that overall dynamics of this protein structure is similar to that of 2b23.

#### Preparation of systems for energy minimization

Structures were solvated with the gmx solvate module of GROMACS 5.0.2^62^ in a dodecahedral box with box edges 1 nm from the solute. Missing residues of 2b23 (except the C-terminal region) were not modelled. The box was filled with explicit TIP3P waters^63^. Parameters from the Amber99SB-ILDN^64^ force field were used. Sodium or chloride counter ions were added to neutralize the system. The N-terminal region of the receptor proteins was capped; the co-activator peptide was modelled with charged termini.

#### Energy minimization

The optimization of the simulation boxes prior MD and docking calculations were done in two steps. This procedure was applied for all cases. In the first step a steepest descent minimization was performed on the solvated box, with convergence threshold set to 10^3^ kJmol^−1^nm^−1^. It was followed by a conjugate gradient minimization, in this step, the convergence was set to 10 kJmol^−1^nm^−1^. Position restraints were applied on solute heavy atoms at a force constant of 10^3^ kJmol^−1^nm^−2^ in both steps.

#### Molecular dynamics (MD)

After minimization, prior to the productive GROMACS MD calculations, a uniform equilibration procedure was performed. The optimized structure was equilibrated under NPT conditions for 10 ns (with 2 fs time step). The solvent and the solute was coupled separately to 300 K with the velocity-rescaling algorithm^65^, with time constant of 0.1 ps. Pressure was kept at 1 bar with the Berendsen barostat^66^ with time constant of 0.5 ps, and compressibility of 4.5 × 10^–5^ bar^−1^. Long range interactions were cut off at 1.1 nm. Position restraints of 1000 kJmol^−1^nm^−1^ were applied on all protein heavy atoms. After equilibration, productive NPT MD calculations were started using GROMACS, with position restraints removed. Pressure was coupled with the Parrinello-Rahman barostat^67^ with time constant of 0.5 ps, and compressibility of 4.5 × 10^−5^ bar^−1^. The temperature was coupled to 300 K with the velocity-rescaling algorithm^65^, with time constant of 0.1 ps, with solvent and solute coupled separately. Coordinates were saved at regular time-intervals, at every 10 ps. Simulation on the ligand free structures were 1 μs-long, the terminal loop was simulated for 100 ns. Periodic boundary conditions were treated after the finish of the calculations.

#### Evaluation of MD results

A ligand free simulation of 1µs length contains 10^5^ frames. RMSF calculation was performed with GROMACS gmx rmsf program. RMSF values of 462–471 and 297–300 residues in Fig. 2c were obtained from simulations with 3q95 (Supplementary Fig. S5). Distance calculations from the initial position of M357 (SD), H356 (CE1) and L327 (CG) sidechain atoms was followed throughout the 1µs steroid-free simulation. The distance was calculated using GROMACS rms program, having an alignment of heavy atoms on the initial structure, over H3 (341–361) and H12 (539–545) residues. For efficient presentation in Fig. 3 (bottom part) and Supplementary Fig. S6 average distances were plotted for every 200 frames.

### Binding site search with blind docking

#### Preparation of the target

The most populous cluster from the 3q95 simulation after 100 ns with the modelled C-terminal was used as the target structure. Clustering was performed with Gromacs program cluster using the gromos method, and a 2 Å cut-off RMSD was set between clusters. Only polar hydrogens were treated explicitly, non-polar hydrogens were merged. Gasteiger-Marsili charges^68^ were added to the protein.

#### Preparation of the ligand

The first step was a steepest descent optimization, with 10^4^ steps. The next step was a conjugate gradient minimization, with a maximum of 10^4^ steps, the with convergence threshold set to 10^−7^ kcalmol^−1^Å^−1^. MMFF94 force field^69^ was used in both steps. The third and last step was performed on semi-empirical quantum mechanical level with MOPAC2012^70^ with PM7 parametrization^71^. Gradient norm was set to 0.01 kcalmol^−1^Å^−1^. After optimization, force calculations were carried out, ensuring that in all cases, the force constant matrices were positive definite. This optimized structure was used in the dockings with Gasteiger-Marsili charges added.

#### Calculation of grid maps

The grid box around the protein was generated with AutoGrid 4.2^72^. The box was centred to cover the whole protein with 200 grid points along all axes, with a spacing of 0.375 Å.

#### Blind docking

Blind docking calculations^52–54^ of the two steroids (E2 and EN) were performed. Docking calculations were performed with AutoDock 4.2.3^72^, Lamarckian genetic algorithm with Solis-Wets local search was used in geometrical search. Dockings started with a population size of 250, the number evaluations were 10^7^, and the number of generations was set to 10^7^. 100 runs were performed in one docking. For RMSD calculation, between the crystallized and the docked estradiol, 1gwr hHERα was used, where estradiol is the co-crystallized ligand (Supplementary Fig. S1). The estradiol structure from 1gwr was taken after superimposing the Cα atoms of 1gwr on to the Cα atoms of hERα structure used for docking.

#### Calculation of interaction energy (E_inter_)

Calculation E_inter_ between docked steroids and hERα (Section Binding sites of sexual steroids) was performed after energy minimization with Gromacs (see previous Section, Energy minimization) of the docked complexes. A Lennard-Jones potential (Equation 2) was used with Amber parameters^73^.

2 $${{\rm{E}}}_{{\rm{inter}}}=\sum _{{\rm{i}},{\rm{j}}}^{{{\rm{N}}}_{{\rm{T}}}{{\rm{N}}}_{{\rm{L}}}}(\frac{{{\rm{A}}}_{{\rm{ij}}}}{{{\rm{r}}}_{{\rm{ij}}}^{12}}\,-\,\frac{{{\rm{B}}}_{{\rm{ij}}}}{{{\rm{r}}}_{{\rm{ij}}}^{6}})$$

$${{\rm{A}}}_{{\rm{ij}}}={{\rm{\varepsilon }}}_{{\rm{ij}}}{{\rm{R}}}_{{\rm{ij}}}^{12}$$

$${{\rm{B}}}_{{\rm{ij}}}=2{{\rm{\varepsilon }}}_{{\rm{ij}}}{{\rm{R}}}_{{\rm{ij}}}^{6}$$

$${{\rm{R}}}_{{\rm{ij}}}={{\rm{R}}}_{{\rm{i}}}+{{\rm{R}}}_{{\rm{j}}}$$

$${{\rm{\varepsilon }}}_{{\rm{ij}}}=\sqrt{{{\rm{\varepsilon }}}_{{\rm{i}}}{{\rm{\varepsilon }}}_{{\rm{j}}}}$$

where N_T_: number of target atoms, N_L_: number of ligand atoms, r_ij_: actual inter-nuclear distance, ε_ij_ = potential well depth at equilibrium between i and j atoms types combined from individual well depths, R_ij_ = inter-nuclear distance at equilibrium between i and j atom types combined from individual radii.

### Interaction dynamics of steroid-bound systems

#### Molecular dynamics

The conditions of MD simulations were the same as described at the steroid-free calculations, except that the present steroid-bound trajectories were 100-ns-long each, and 1001 frames were sampled per trajectory. After each trajectory the periodic boundary effects were handled, the system was centred in the box and target molecules in subsequent frames were fit on the top of the first frame. In order to compare the “Open” and the “Closed” state between each other (Fig. 7a), after handing the periodic boundary effects, the first frame of “Open” state was superposed on the “Closed” state by their Cα atoms.

#### Kinetic stability

Residence frequency (RF, Equation 3) was calculated as a measure of kinetic stability. The movement of the ligand was described by the distance between the centre of mass (d_COM_) of its actual and starting positions at each time frame during the total simulation time.

3 $${\rm{RF}}=\frac{{\rm{Count}}\,{\rm{of}}\,{\rm{time}}\,{\rm{frames}}\,{\rm{with}}\,{{\rm{d}}}_{{\rm{COM}}}\le \,{{\rm{d}}}_{{\rm{LIM}}}}{{\rm{Simulation}}\,{\rm{time}}\,({\rm{ns}})}$$

The value of dissociation limit d_LIM_ was set to 5 Å. The RF values were calculated for the five individual trajectories and also for a merged trajectory of 500 ns including all five trajectories. The theoretical upper limit of RF was 10.0 ns^−1^ (=1001/100 ns) in the present study which corresponds to the highest kinetic stability.

#### Correlation of movements of M357, H356, L327 residues, with dissociation of EN

The distance of the side chain atoms from the initial positions were calculated using Gromacs rms program, having an alignment of heavy atoms, on the initial structure, over H3 (341–361) and H12 (539–545) residues. Using the same technique as in the steroid-free evaluations (Methods), for efficient presentation, average distance values were calculated for every 10 frames, resulting in 100 distances for 100 ns of simulation. Correlation of movements of M357, H356, L327 residues, with dissociation of EN was followed when d_COM ≤ _d_LIM_. The d_LIM_ corresponded to 27.2 ns, and correlation points (Fig. 6), were taken from 0 to 28 ns. Average distances of the investigated time interval (0–28 ns) resulted in 1.2 Å initial, and 4.9 Å final d_COM_ values, shown as abscissa in Fig. 6. Up until this frame, the ligand dissociation could be correlated with all three residues, and this is also the point when EN starts to abruptly dissociate from ABS (Fig. 5). The structural representations in Fig. 6, were taken from 18 ns. This was the frame when the movement of all three residues was the most representative.

#### Velocity calculations

In order to characterize the dissociation patterns of both E2 and EN, three types of velocities were calculated, and presented in Supplementary Table S6–7. The v_1_ measures the ligand velocity in the initial dissociation phase, until d_LIM_ is reached. The second type of velocity (v_2_) takes into account the necessary time for the ligand to reach total dissociation after reaching the d_LIM_. The limit for final dissociation was set to 10 Å, and the time when this limit is reached, was collected in Supplementary Table S6. The v_2_ characterizes the best, the differences between EN and E2 dissociation mode. In CA− simulations, d_LIM_ was not was not reached for E2, and therefore, v_2_ was not calculated. The third type of velocity (v_3_) describes the ligand velocity for the total dissociation, from the start of the simulation.

### Data availability statement

The datasets generated during and/or analysed during the current study are included in this published article (and its Supplementary Information files) or available from the corresponding author on reasonable request.

## Electronic supplementary material

Supplementary Information

The three-dimensional structure of the hERα

An example 100-ns-long molecular dynamics (MD) simulation

The three-dimensional structure of the flickering gate
