# Supplementary material for: Dynamic changes in binding interaction networks of sex steroids establish their non-classical effects
Source: Sci Rep. 2017 Nov 1;7:14847. doi: 10.1038/s41598-017-14840-9 (PMC5665952; doi:10.1038/s41598-017-14840-9)
Supplement: Supplementary file 1 — Supplementary Information [file 41598_2017_14840_MOESM1_ESM.pdf]

Supplementary Information

Dynamic changes in binding interaction networks of sex steroids establish their non-classical effects

Mónika Bálint[1,2,†], Norbert Jeszenői[3,†], István Horváth[4], István M. Ábrahám[3,], Csaba Hetényi[1,*]*

*Corresponding authors.
istvan.abraham@aok.pte.hu
csabahete@yahoo.com

[†]Equal contribution.

[1]Department of Pharmacology and Pharmacotherapy, University of Pécs, Szigeti út 12, 7624 Pécs, Hungary.
[2]Department of Biochemistry, Eötvös Loránd University, Pázmány Péter sétány 1/C, 1117 Budapest, Hungary.
[3]MTA NAP-B Molecular Neuroendocrinology Group, Institute of Physiology, Szentágothai Research Center, Center for Neuroscience, University of Pécs, Szigeti út 12, 7624 Pécs, Hungary.
[4]Chemistry Doctoral School, University of Szeged, Dugonics tér 13, 6720 Szeged, Hungary.

Supplementary Figures

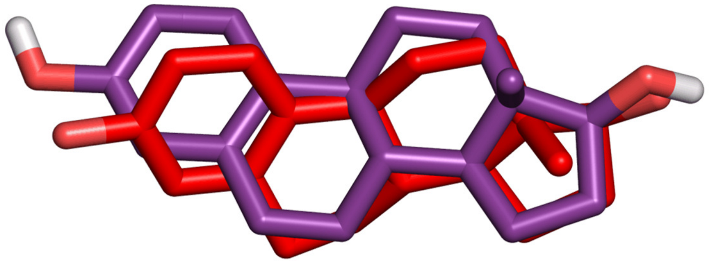

Supplementary Figure S1 Conformation of E2 obtained with blind docking (magenta) compared to the reference structure (red), from X-ray structure (1gwr).

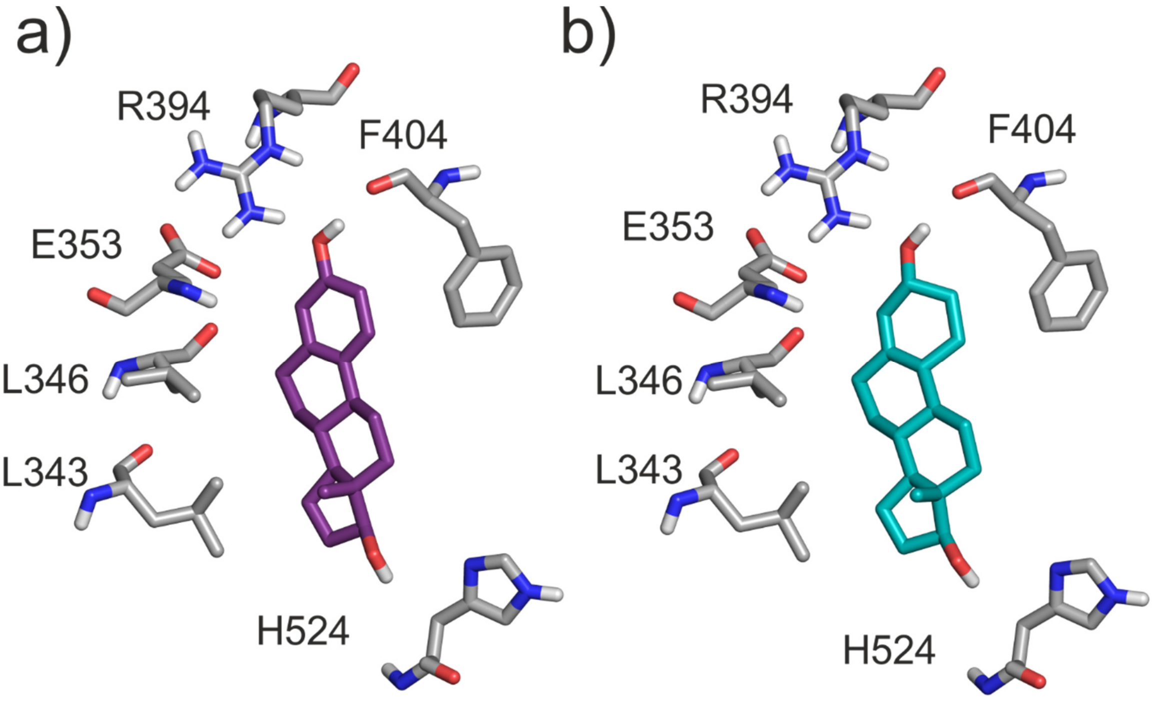

Supplementary Figure S2 Binding conformations of E2 (a, magenta) and EN (b, teal) in CBS are presented. The main interacting amino acids are in sticks with grey carbon atoms. In contrast with ABS (Fig. 4, main text), the two molecules (E2 and EN) bind to CBS in a similar orientation.

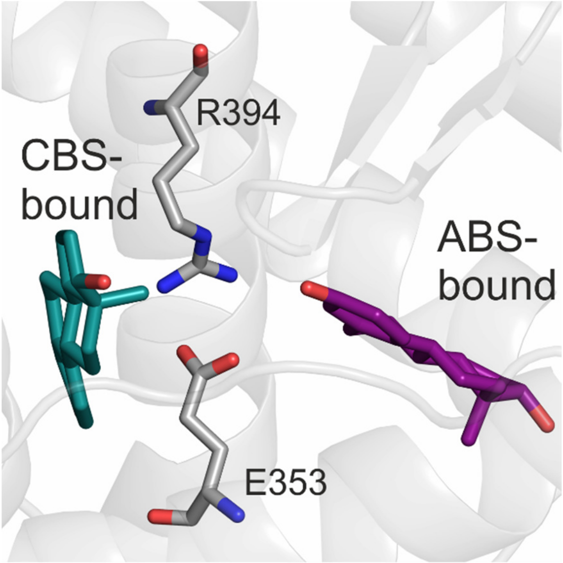

Supplementary Figure S3 Docked conformations of E2 are presented in magenta (ABS) and teal (CBS). The two binding sites are separated by R394 and E353.

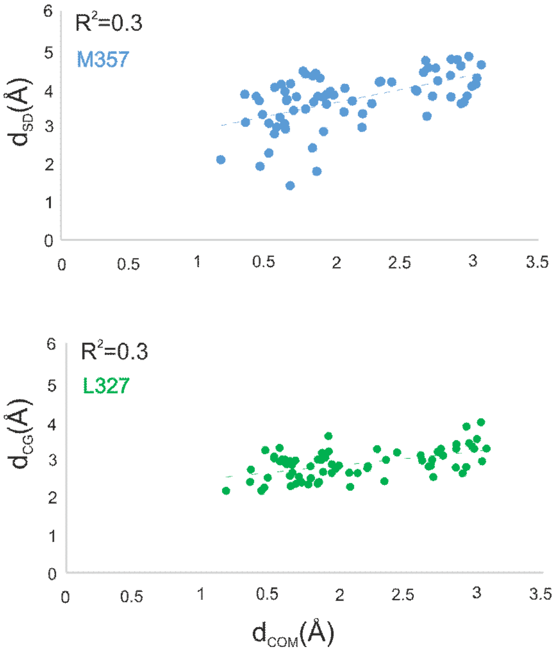

Supplementary Figure S4 Correlations observed in simulations with EN, case CA-. Similarly to the main text, points were taken until the ligand reached the d_{LIM} dissociation criteria, which was 70 ns in this case. Correlations are plotted for the movements of L327 and H357. Although the correlation is not as strong as observed in CA+ scenario described in the main text, the role of both M357 and L327 appears to be important in ligand dissociation, even without the presence of CA.

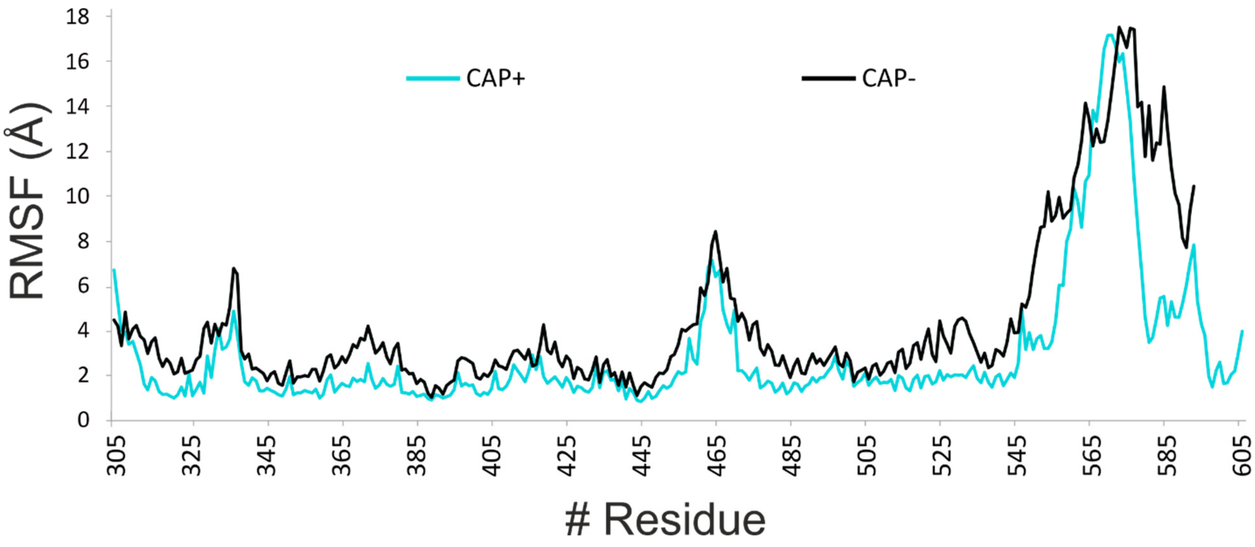

Supplementary Figure S5 RMSF of 3q95 during 1μs ligand-free CA- and CA + simulations. RMSF was calculated and presented in the same way as in Fig. 3 of the main text. Similarly to Fig. 3, high RMSF values were observed in L1, L2 and in the F domain. The above-presented similarities in the RMSF between 3q95 and 2b23 demonstrates that the two crystallographic structures have a similar dynamic behaviour in additon to their excellent structural alignement of 0.5 Å (Methods).

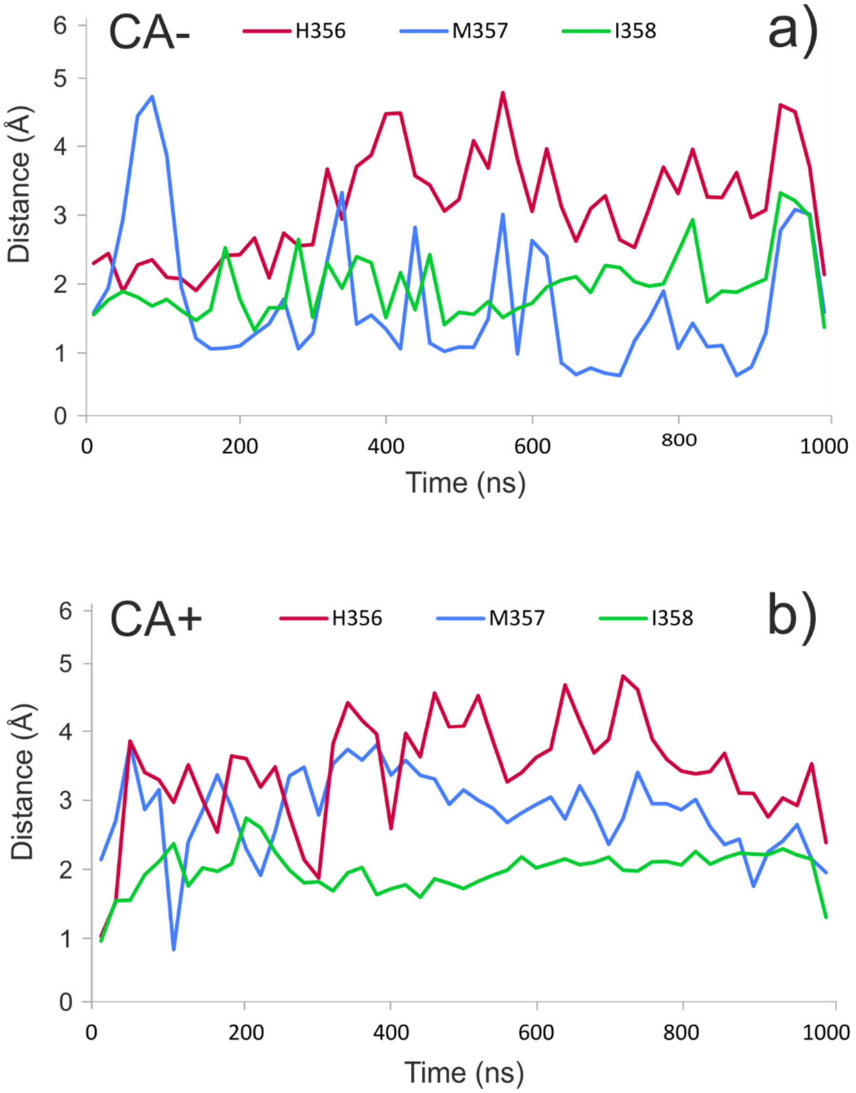

Supplementary Figure S6 Distances of actual positions of M357 (SD), H356 (CE1), and I358 (CG) sidechain atoms calculated from their initial positions during ligand-free simulations CA- (a) and CA+ (b) (PDB structure 3q95). The calculations were performed in the same way as presented in Methods of the main text. In the CA- simulations (a), it can be observed that H356 is the most mobile residue among the three analyzed, I358 and M357 have high mobility explained by the absence of the restraining factor CA (see main text, Section 1). In CA+ (b) simulations, the mobility of I358 decreases due to the prescence of CA, similarly to Fig. 3 and its distance from the initial position remains constant. On the other hand, an increase in the distances is observed for H356 and M357, the two amino acids that switch inside the ABS, in the presence of CA. The above observations support the proposed mechanism and the observations presented in the main text (Fig. 3).

Supplementary Tables

Supplementary Table S1 Ranks obtained from blind docking of E2 and EN to hERα

#Rank	$\Delta G_{scoring}$(kcal/mol)*	
	E2	EN
1	-8.16	-8.75
2	-7.56	-8.1
3	-6.69	-6.56
4	-6.54	-6.56
5	-6.18	-6.07
6	-6.15	-5.66
7	-5.96	-
8	-5.67	-
9	-5.64	-
10	-5.01	-
11	-4.88	-

*The ranks are ordered by their AutoDock 4.2 scoring values.

Supplementary Table S2 Residence frequency values for EN calculated using RMSD

CA	+		-		+	-	-	-
ABS	+		+		+		-	
CBS	+		+		-		+	
seed1	**2.7**	**10.0**	10.0	10.0	5.6	1.9	**10.0**	10.0
seed2	0.8	6.6	2.6	10.0	1.7	**6.2**	10.0	1.8
seed3	3.6	10.0	7.6	10.0	6.7	3.2	10.0	**10.0**
seed4	1.4	10.0	**7.0**	**10.0**	**6.4**	6.0	5.5	10.0
seed5	2.0	10.0	6.5	10.0	10.0	2.3	10.0	10.0
all seeds	2.1	9.3	6.7	10.0	6.1	3.9	9.1	8.4

*Bold numbers represent the simulations with RF values closest to the average RF.

Supplementary Table S3 Residence frequency values for EN calculated using d_{COM}

CAP	+		-		+	-	+	-
ABS	+		+		+		-	
CBS	+		+		-		+	
seed1	**2.7**	**10.0**	10.0	10.0	6.6	2.3	**10.0**	10.0
seed2	0.8	6.6	3.8	10.0	6.3	**6.3**	10.0	1.8
seed3	5.9	10.0	7.6	10.0	10.0	9.6	10.0	**10.0**
seed4	1.5	10.0	**7.0**	**10.0**	**7.0**	9.1	5.4	10.0
seed5	2.0	10.0	8.3	10.0	10.0	5.1	10.0	10.0
all seeds	2.6	9.3	7.2	10.0	8.0	6.5	9.1	8.4

*Bold numbers represent the simulations with RF values closest to the average RF. These simulations were selected as reference, and used to present results in Table 2 and 3 of the main text.

Supplementary Table S4 Residence frequency values for E2 calculated using RMSD

CAP:	+		-		+	-	+	-
ABS	+		+		+		-	
CBS	+		+		-		+	
seed1	0.7	10.0	10.0	10.0	0.9	0.1	**10.0**	**10.0**
seed2	0.7	10.0	10.0	10.0	**2.2**	4.1	10.0	10.0
seed3	0.2	10.0	0.3	10.0	0.2	0.2	10.0	10.0
seed4	**1.1**	**10.0**	**2.3**	**10.0**	0.1	**1.4**	10.0	10.0
seed5	1.3	10.0	0.7	10.0	0.1	0.1	10.0	10.0
all seeds	0.8	10.0	4.6	10.0	0.7	1.2	10.0	10.0

*Bold numbers represent the simulations with RF values closest to the average RF.

Supplementary Table S5 Residence frequency values for E2 calculated using d_{COM}

CAP:	+		-		+	-	+	-
ABS	+		+		+		-	
CBS	+		+		-		-	
seed1	1.03	10.0	10.0	10.0	8.7	0.1	**10.0**	**10.0**
seed2	0.74	10.0	10.0	10.0	**5.3**	9.5	10.0	10.0
seed3	3.56	10.0	2.2	10.0	0.3	0.2	10.0	10.0
seed4	**1.74**	**10.0**	**2.7**	**10.0**	0.1	**1.7**	10.0	10.0
seed5	1.72	10.0	0.8	10.0	0.1	0.1	10.0	10.0
all seeds	1.76	10.0	5.1	10.0	2.9	2.3	10.0	10.0

*Bold numbers represent the simulations with RF values closest to the average RF. These simulations were selected as reference, and used to present results in Table 2 and 3 of the main text.

Supplementary Table S6 Simulation times corresponding to $d_{COM} \geq 5$ Å and $d_{COM} \geq 10$ Å values

d_{COM}	Time (ns)				
		EN		E2	
		CA+	CA-	CA+	CA-
5	t_2	27.2	70.3	4.1	6.2
10	t_3	40.0	89.9	96.7	-

Supplementary Table S7 Velocity (Å/ns) values of EN and E2, during CA-/ABS+/CBS+ and CA+/ABS+/CBS+ simulations

Velocity (Å/ns)					
		EN		E2	
v	Time interval	CA+	CA-	CA+	CA-
v_1	t_2-t_1	0.18	0.07	1.22	0.81
v_2	t_3-t_2	0.39	0.26	0.10	-
v_3	t_3-t_1	0.25	0.11	0.05	-

Supplementary Table S8 List of hERα structures in the PDB

PDB code	Method	Resolution(Å)	Residues
1A52	X-ray	2.8	297-554
1AKF	model	-	309-547
1ERE	X-ray	3.1	301-553
1ERR	X-ray	2.6	301-553
1G50	X-ray	2.9	304-550
1GWQ	X-ray	2.45	301-548
1GWR	X-ray	2.4	305-549
1HCP	NMR	-	180-254
1HCQ	X-ray	2.4	180-262
1L2I	X-ray	1.95	297-554
1PCG	X-ray	2.7	304-547
1QKT	X-ray	2.2	304-551
1QKU	X-ray	3.2	301-550
1R5K	X-ray	2.7	297-554
1SJ0	X-ray	1.9	307-554
1UOM	X-ray	2.28	301-553
1X7E	X-ray	2.8	305-549
1X7R	X-ray	2	305-549
1XP1	X-ray	1.8	307-554
1XP6	X-ray	1.7	307-554
1XP9	X-ray	1.8	307-554
1XPC	X-ray	1.6	307-554
1XQC	X-ray	2.05	301-553
1YIM	X-ray	1.9	307-554
1YIN	X-ray	2.2	307-554
1ZKY	X-ray	2.25	298-554
2AYR	X-ray	1.9	304-551
2B1V	X-ray	1.8	298-554
2B1Z	X-ray	1.78	298-554
2B23	X-ray	2.1	298-554
2BJ4	X-ray	2	305-533
2FAI	X-ray	2.1	298-554
2G44	X-ray	2.65	298-554
2G5O	X-ray	2.3	298-554
2I0J	X-ray	2.9	304-547
2IOG	X-ray	1.6	309-554
2IOK	X-ray	2.4	301-554
2JF9	X-ray	2.1	304-533
2JFA	X-ray	2.55	304-533

PDB code	Method	Resolution(Å)	Residues
2LLO	NMR	-	287-305
2OCF	X-ray	2.95	298-595
2OUZ	X-ray	2	301-553
2P15	X-ray	1.94	298-554
2POG	X-ray	1.84	304-551
2Q6J	X-ray	2.7	298-554
2Q70	X-ray	1.95	304-551
2QA6	X-ray	2.6	298-554
2QA8	X-ray	1.85	298-554
2QAB	X-ray	1.89	298-554
2QE4	X-ray	2.4	304-551
2QGT	X-ray	2.15	298-554
2QGW	X-ray	2.39	298-554
2QH6	X-ray	2.7	298-554
2QR9	X-ray	2	298-554
2QSE	X-ray	1.85	298-554
2QXM	X-ray	2.3	298-554
2QXS	X-ray	1.7	298-554
2QZO	X-ray	1.72	298-554
2R6W	X-ray	2	304-551
2R6Y	X-ray	2	304-551
2YAT	X-ray	2.6	301-551
2YJA	X-ray	1.82	299-551
3CBM	X-ray	1.69	298-307
3CBO	X-ray	1.65	298-307
3CBP	X-ray	1.42	298-307
3DT3	X-ray	2.4	299-551
3ERD	X-ray	2.03	297-554
3ERT	X-ray	1.9	297-554
3HLV	X-ray	3	298-550
3HM1	X-ray	2.33	298-550
3L03	X-ray	1.9	298-550
3OS8	X-ray	2.03	299-553
3OS9	X-ray	2.3	299-553
3OSA	X-ray	2.3	299-553
3Q95	X-ray	2.05	298-554
3Q97	X-ray	2.1	298-554
3UU7	X-ray	2.2	302-552
3UUA	X-ray	2.05	302-552
3UUC	X-ray	2.1	302-552
3UUD	X-ray	1.6	302-552

PDB code	Method	Resolution(Å)	Residues
4AA6	X-ray	2.6	182-252
4DMA	X-ray	2.3	303-549
4IU7	X-ray	2.29	303-549
4IUI	X-ray	2.3	303-549
4IV2	X-ray	2.14	303-549
4IV4	X-ray	2.3	303-549
4IVW	X-ray	2.06	303-549
4IVY	X-ray	1.95	303-549

Supplementary Video legends

Supplementary Video 1

The three-dimensional structure of the hERα, with important structural elements highlighted in red. ABS and CBS binding sites are also presented in the video.

Supplementary Video 2

An example 100-ns-long molecular dynamics (MD) simulation of EN dissociation from the ABS. The simulation started from a CA+/CBS+/ABS+ starting complex (Fig. 1). Disruption of H-bonds with K449, and L327 can be observed at 25 and 30 ns, respectively.

Supplementary Video 3

The three-dimensional structure of the flickering gate is composed by T1, L1, and H3 structural elements. Its closed (blue) and opened (red) states show considerable differences at T1 and L1 conformations. EN is presented with red (ABS-bound) and blue (unbound) sticks.
